# Oncological outcomes of robotic-assisted versus open pancreatoduodenectomy for pancreatic ductal adenocarcinoma: a propensity score-matched analysis

**DOI:** 10.1007/s00464-020-07791-2

**Published:** 2020-07-21

**Authors:** Yuanchi Weng, Yu Jiang, Ningzhen Fu, Jiabin Jin, Yusheng Shi, Zhen Huo, Xiaxing Deng, Chenghong Peng, Baiyong Shen

**Affiliations:** Department of General Surgery, Pancreatic Disease Center, Ruijin Hospital, Shanghai Jiao Tong University School of Medicine, No. 197 Ruijin Er Road, Huangpu District, Shanghai, 200025 China

**Keywords:** Pancreatic ductal adenocarcinoma, Robotic-assisted pancreatoduodenectomy, Open pancreatoduodenectomy, Propensity score matching

## Abstract

**Background:**

Robotic-assisted minimally invasive surgery is associated with worse oncologic outcomes for some but not other types of cancers. We conducted a propensity score-matched analysis to compare oncologic outcomes of robotic-assisted laparoscopic (RPD) *vs.* open pancreatoduodenectomy (OPD) for pancreatic ductal adenocarcinoma (PDAC).

**Methods:**

Treatment-naïve PDAC patients undergoing either RPD or OPD at our hospital between January 2013 and December 2017 were included. Propensity score matching was conducted at a ratio of 1:2. The primary outcome was disease-free survival (DFS) and overall survival (OS).

**Results:**

A total of 672 cases were identified. The propensity score-matched cohort included 105 patients receiving RPD and 210 patients receiving OPD. The 2 groups did not differ in the number of retrieved lymph nodes [11 (7–16) *vs.* 11 (6–17), *P* = 0.622] and R0 resection rate (88.6% *vs*. 89.0%, *P* = 0.899). There was no statistically significant difference in median DFS (14 [95% CI 11–22] *vs.* 12 [95% CI 10–14] months (HR 0.94; 95% CI 0.87–1.50; log-rank *P* = 0.345) and median OS (27 [95% CI 22–35] *vs.* 20 [95% CI 18–24] months (HR 0.77; 95% CI 0.57–1.04; log-rank *P* = 0.087) between the two groups. Multivariate COX analysis showed that RPD was not an independent predictor of DFS (HR 0.90; 95% CI 0.68–1.19, *P* = 0.456) or OS (HR 0.77; 95% CI 0.57–1.05, *P* = 0.094).

**Conclusion:**

Comparable DFS and OS were observed between patients receiving RPD and OPD. This preliminary finding requires further confirmation with prospective randomized controlled trials.

Open pancreatoduodenectomy (OPD) is the cornerstone in the treatment of pancreatic ductal adenocarcinoma (PDAC) in the pancreatic head or uncinate process, but is associated with significant morbidities [1–4]. Minimally invasive surgery was introduced in 1994 for PDAC patients [5], followed by robotic-assisted pancreatoduodenectomy (RPD) in 2003 [6]. Though technically challenging, RPD has a variety of advantages, including less blood loss, faster recovery, and less postoperative complications [4, 7–12]. However, the long-term oncological outcomes of RPD remain undefined [13–17].

Propensity score matching is a statistical method to minimize bias in retrospective studies [18]. We conducted a retrospective analysis to compare the long-term oncological outcomes between RPD and OPD using propensity score-matching. The primary end point of the study was disease-free survival (DFS) and overall survival (OS).

## Methods

The study was undertaken according to the Strengthening the Reporting of Observational Studies in Epidemiology (STROBE) guidelines [19] and in accordance with the latest version of the Declaration of Helsinki. The study protocol was approved by the Institutional Review Board, Ruijin Hospital. Informed consent was waived since the study was observational and retrospective.

### Patient selection and treatment

We conducted this retrospective propensity score matched cohort study from a prospective database, and included treatment-naïve PDAC patients who underwent RPD or OPD between January 2013 and December 2017 at the Pancreatic Surgery Department of Ruijin Hospital affiliated to Shanghai Jiaotong University School of Medicine. The RPD and OPD cases included in this study were performed by the same group of surgeons, who had experience of OPD for more than 1000 cases and experience of laparoscopic pancreatic surgery for more than 120 cases. The selection of RPD was based on our surgical team’s suggestion, we provided robotic approach choice for the following patients: (1) Stage I or II PDAC cases without “borderline resectable” lesions; (2) Preoperative serum total bilirubin ≤ 250 μmol/L; (3) Patients less than 90 years old; (4) ASA score I–III; (5) Without complicated major abdominal surgery history; (6) Without contraindication of pneumoperitoneum. Finally, patients made their choice according to their preference and acceptance of the robotic approach. Our initial cases of RPD (from 2010 to 2012), as cases surpassing the learning curve according to previous study about the learning curve of RPD, were not included [20]. The diagnosis of PDAC was established according to the NCCN Guidelines for Pancreatic Adenocarcinoma on the basis of differential diagnosis to exclude mucous cystadenocarcinoma, signet ring cell carcinomas, adenosquamous carcinomas, undifferentiated (anaplastic) carcinomas, and mixed ductal-endocrine carcinomas [21]. PDAC was staged based on the AJCC 8th TNM stage manual [22]. Major exclusion criteria were: (1) heterogeneous carcinoma (e.g., intraductal papillary mucinous tumor or pancreatic adenosquamous carcinoma), (2) T4 and/or M1 disease, and (3) missing key clinical variables or follow-up data.

After passing the learning curve of RPD, our surgical team concluded a standard surgical process. The detailed surgical procedures and lymphadenectomy are described in our previous study [23]. After laparoscopic exploration, if diagnosed as T4 or M1 stage, curative surgery would not been performed, and patients would receive subsequent chemotherapy. The “artery first” approach was used when tumors were adjacent to superior mesenteric artery and when portal vein (PV) or superior mesenteric vein (SMV) were involved. PV/SMV wedge resection and repair or end-to-end anastomosis would be performed in PV/SMV involved cases.

Postoperative adjuvant chemotherapy included multi-agent gemcitabine-based, single-agent gemcitabine based and folfirinox regimens. Patients were followed up in out-patient department or by telephone contact with the patients or their families every 3 months. Post recurrence chemotherapy represented chemotherapy after recurrence was diagnosed, regardless of adjuvant chemotherapy, also with multi-agent gemcitabine-based, single-agent gemcitabine based and folfirinox regimens.

### Definitions and data collection

Preoperative variables were retrieved from the hospital’s electronic records system, including age, sex, body mass index (BMI), previous abdominal surgery history, preoperative biliary drainage, total bilirubin, CA19-9 and American Society of Anesthesiologists (ASA) physical status [24], biliary drainage included preoperative biliary stent placement, nasobilliary drainage and percutaneous transhepatic cholangial drainage.

We also obtained data on tumor size defined by the longest diameter of the primary tumor, number of retrieved lymph node, positive lymph nodes with cancer cell metastasis, lymph node ratio which calculated as the number of positive lymph nodes divided by the number of retrieved lymph nodes, TNM stage, R0 resection, lymphovascular invasion and perineural invasion. R0 resection was defined as absence of malignant cells within 1 mm from the resection margin using the Royal College of Pathologists definition [25]. Lymphovascular invasion and perineural invasion were based on pathologic report using paraffin sections.

Intraoperative and postoperative variables were also recorded. For RPD cases, docking time was included in the calculation of operative time, estimated blood loss was evaluated based on the vacuum amount, gauze weight and intraoperative fluid infusion volume. Postoperative pancreatic fistula, postpancreatectomy hemorrhage, and delayed gastric emptying represented complications defined by International Study Group on Pancreatic Surgery [1–3]. Biliary fistula was diagnosed by positive drainage of bile acid. Anastomosis fistula was diagnosed by contrastography. Surgical site infections were defined by the Center for Disease Control and Prevention (CDC) definition [26–28], diagnosed by positive pathogen culture in 2 weeks from surgery. Other complications were classified as Clavien–Dindo grade ≥ 3 other than the complications listed above [29].

Follow-up data through September 2019 was retrieved, DFS was calculated from the date of surgery to the date of recurrence or last follow-up if recurrence did not occur. Recurrence was diagnosed by CT or MRI imaging examination. OS was defined as the time from surgery to either death or last follow-up. Patients with death attributed to perioperative morbidity within 90 days or other non-disease-specific reasons in the postoperative period were also censored, only disease-specific recurrence and disease-specific death were defined as end point events.

### Matching

According to previous reports about the important factors associated with the short-term and long-terms outcomes, together with the variables that would affect the outcomes of RPD and OPD, the propensity score was calculated based on the covariates age, sex, BMI, abdominal surgery history, ASA physical status, CA199, total bilirubin, biliary drainage, tumor size, portal-mesenteric vein resection, year of diagnosis, differentiation, T and N stage, lymphovascular invasion and perineural invasion and adjuvant chemotherapy. RPD cases, regardless of conversion to laparotomy PD, were matched to OPD group in a 1:2 ratio and a caliper width of 0.05 standard deviation (SD) was specified.

### Statistical analysis

IBM SPSS Statistics version 24.0 (IBM Corporation) and the statistical packages R (The R Foundation; https://www.r-project.org; version 3.4.3) were used for statistical analysis. Normally distributed continuous variables were presented as mean and SD, and analyzed using Student’s *t*-test. Non-normally distributed continuous variables are presented as median and interquartile range (IQR), and analyzed using the Mann–Whitney *U* test. Categorical variables were presented as frequency or percentage, and analyzed using Chi-square test or Fisher's exact test. Survival analysis of OS and DFS and their corresponding 95% confidence intervals (CIs) was plotted by the Kaplan–Meier curved and compared with log rank test. Univariate analysis and multivariate COX model analysis were undertaken. When continuous variables were converted to categorical variables, the cutoffs were defined by what previously reported in the literature or by the ROC curve, and variables with *P* value < 0.1 in univariate analysis were included into multivariate Cox proportional hazards model for multivariable analysis. *P* < 0.05 was considered statistically significant.

## Results

### Demographic and baseline characteristics of the study population

**Fig. 1:**
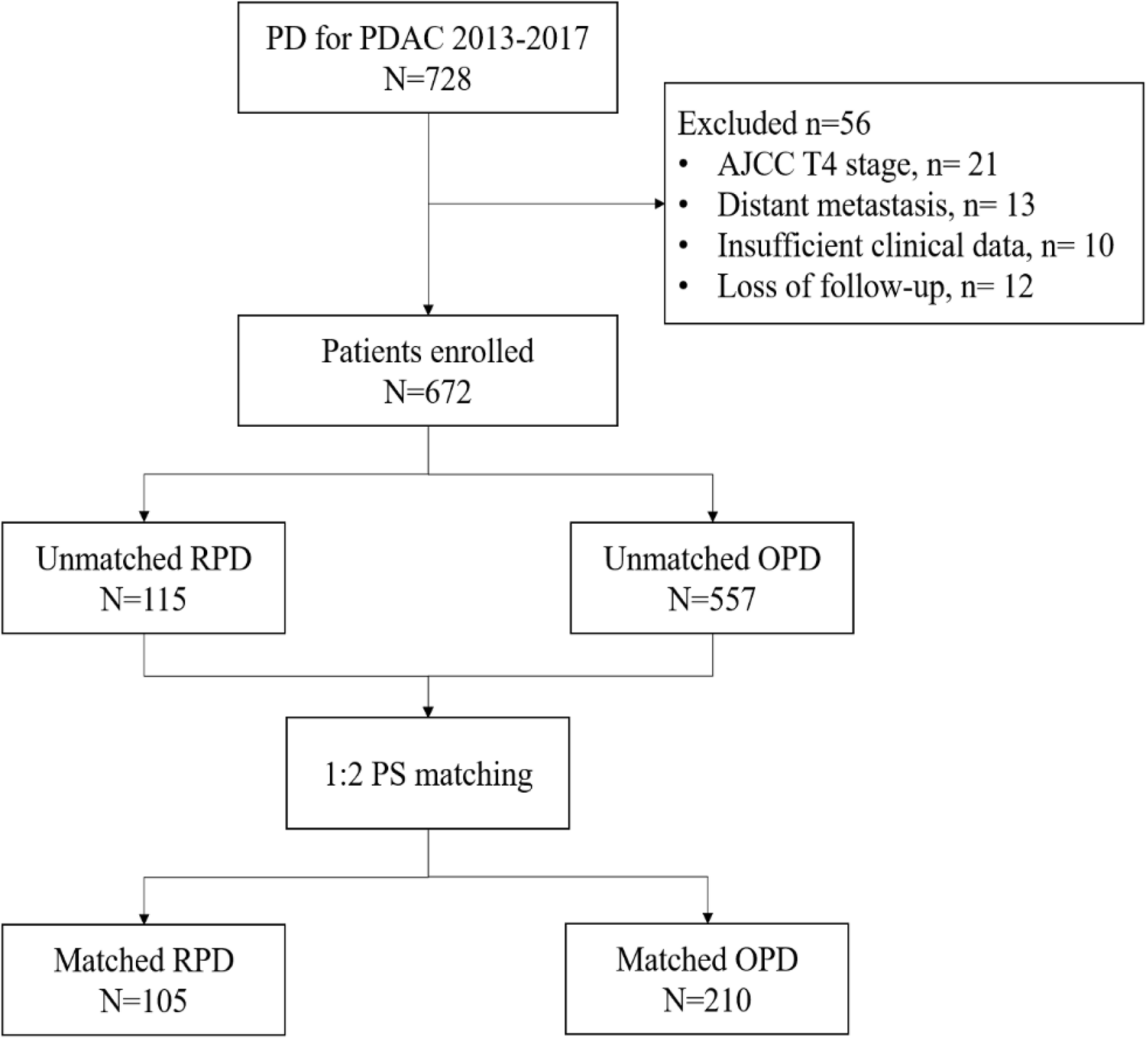
The study flowchart

A total of 728 patients underwent RPD or OPD for PDAC during the study period (Fig. 1). 56 were excluded from the final analysis for the following reasons: T4 disease (*n* = 21), distant metastasis (*n* = 13), incomplete data (*n* = 10), and loss to follow up (*n* = 12). The final analysis included 672 subjects (438 male patients, 234 female patients; age at diagnosis: 64 [58–70] years). 115 patients underwent RPD and 557 patients underwent OPD. The demographic and baseline characteristics are presented in Table 1. Compared to the OPD group, the RPD group had significantly lower rate of previous abdominal surgery (7.0% *vs.* 13.5%, *P* = 0.032), lower preoperative total bilirubin (median [IQR] 29.9 [14.4–94.7] *vs.* 64.7 [15.8–154.9] μmol/L, *P* < 0.001), and higher rate of lymphovascular invasion (58.3% *vs.* 47.0%, *P* = 0.028). The T staging was T1-18.3%/T2-51.3%/T3-30.4% for RPD *vs*. T1-19.2%/T2-48.3%/T3-32.5% for OPD (*P* = 0.840). The N staging was N0-57.4%/N1-38.3%/N2-4.3% for RPD *vs*. N0-53.1%/N1-36.3%/N2-10.6% for OPD (*P* = 0.115).

**Table 1:** Demographic and baseline characteristics of the study population

Variables	Total cohort		*P*	Propensity score matched cohort		*P*
	RPD	OPD		RPD	OPD	
N	115	557		105	210	
Age, years, median (IQR)	63 (57–68)	64 (58–70)	0.085	64 (58–58)	62 (58–69)	0.973
Female, *n* (%)	44 (38.3%)	190 (34.1%)	0.395	39 (37.1%)	75 (35.7%)	0.804
BMI, kg/m^2^, mean (SD)	22.8 (2.8)	22.6 (3.1)	0.355	22.7 (2.7)	22.6 (3.0)	0.706
Previous abdominal surgery, *n* (%)	8 (7.0%)	75 (13.5%)	0.032	8 (7.6%)	15 (7.1%)	0.878
TB, μmol/L, median (IQR)	29.6 (14.4–94.7)	64.7 (15.8–154.9)	< 0.001	36.3 (14.9–97.4)	25.4 (13.6–118.2)	0.663
Biliary drainage, *n* (%)	36 (31.3%)	155 (27.8%)	0.452	34 (32.4%)	51 (24.3%)	0.127
ASA score ≥ 3, *n* (%)	21 (18.3%)	94(16.9%)	0.720	19 (18.1%)	44 (21.0%)	0.550
CA199, U/mL, median (IQR)	144.4 (40.1–375.4)	153.4 (46.0–505.2)	0.264	144.4 (38.8–434.5)	116.9 (26.3–404.4)	0.631
Tumor size, cm, median (IQR)	3 (2.2–3.5)	3.0 (2.3–3.8)	0.278	3 (2.5–3.5)	2.5 (2.0–3.6)	0.328
PV/SMV resection, *n* (%)	9 (7.8%)	53 (9.5%)	0.569	9 (8.6%)	20 (9.5%)	0.783
Year of diagnosis, *n* (%)			< 0.001			0.068
2013	3 (2.6%)	29 (5.2%)		3 (2.9%)	6 (2.9%)	
2014	10 (8.7%)	111 (19.9%)		10 (9.5%)	14 (6.7%)	
2015	13 (11.3%)	170 (30.5%)		12 (11.4%)	43 (20.5%)	
2016	36 (31.3%)	117 (21.0%)		36 (34.3%)	46 (21.9%)	
2017	53 (46.1%)	130 (23.3%)		44 (41.9%)	101 (48.1%)	
Poor differentiation, *n* (%)	80 (78.3%)	418 (75.0%)	0.465	80 (76.2%)	158 (75.2%)	0.853
Tumor stage, *n* (%)			0.840			0.388
T1	21 (18.3%)	107 (19.2%)		19 (18.1%)	47 (22.4%)	
T2	59 (51.3%)	269 (48.3%)		53 (50.5%)	89 (42.4%)	
T3	35 (30.4%)	181 (32.5%)		33 (31.4%)	74 (35.2%)	
Lymph node stage, *n* (%)			0.115			0.078
N0	66 (57.4%)	296 (53.1%)		57 (54.3%)	126 (60.0%)	0.333
N1	44 (38.3%)	202 (36.3%)		43 (41.0%)	63 (30.0%)	
N2	5 (4.3%)	59 (10.6%)		5 (4.8%)	21 (10.0%)	
LVI, *n* (%)	67 (58.3%)	262 (47.0%)	0.028	60 (57.1%)	112 (53.3%)	0.522
PNI, *n* (%)	85 (73.9%)	439 (78.8%)	0.248	79 (75.2%)	156 (74.3%)	0.855
Adjuvant chemotherapy, *n* (%)	56 (48.7%)	283 (50.8%)	0.680	54 (51.4%)	108 (51.4%)	1.000

*OPD* open pancreatoduodenectomy, *RPD* robotic pancreatoduodenectomy, *BMI* body mass index, *TB* total bilirubin, *LVI* lymphovascular invasion, *PNI* perineural invasion

After propensity score matching at a ratio of 1:2, 105 patients were included in the RPD group and 210 patients in the OPD group.

### Intraoperative and perioperative characteristics

The intraoperative variables and perioperative outcomes are presented in Table 2. Two patients were converted from RPD to OPD because of bleeding and insufficient surgical view, respectively. After matching, the number of retrieved lymph nodes (RPD: 11 [7–16] *vs*. OPD: 11 [6–17], *P* = 0.622), number of positive lymph nodes (0 [0–1] *vs*. 0 [0–2], *P* = 0.975), operative time, estimated blood loss, and length of postoperative hospital stay and the complication rate were all comparable between the two groups. The 30-day mortality rate for RPD was 0% and 1.0% for OPD (*P* = 0.554). The 90-day mortality rate was 1.0% in both groups.

**Table 2:** Intraoperative and perioperative characteristics of the propensity score matched population

Variables	Total cohort		*P*	Propensity score matched cohort		*P*
	RPD	OPD		RPD	OPD	
*N*	115	557		105	210	
Conversion, *n* (%)	2 (1.7%)	–	–	2 (1.8%)	–	–
Retrieved lymph nodes, median (IQR)	11 (6–16)	12 (7–18)	0.150	11 (7–16)	11 (6–17)	0.622
Positive lymph nodes, median (IQR)	0 (0–1)	0 (0–2)	0.035	0 (0–1)	0 (0–2)	0.975
LNR, median (IQR)	0 (0–0.09)	0 (0–0.14)	0.810	0 (0–0.1)	0 (0–0.1)	0.691
< 0.1	89 (77.4%)	375 (67.3%)		79 (75.2%)	153 (72.9%)	0.082
≥ 0.1	26 (22.6%)	182 (32.7%)		26 (24.8%)	57 (27.1%)	0.516
R0 resection, *n* (%)	103 (89.6%)	486 (87.3%)	0.493	93 (88.6%)	187 (89.0%)	0.899
Blood loss, mL, median (IQR)	300 (200–500)	300 (200–500)	0.746	300 (200–550)	300 (200–500)	0.567
Operative time, min, median (IQR)	300 (245–360)	300 (245–335)	0.606	300 (250–360)	300 (240–330)	0.365
POD, d, median (IQR)	18 (14–25)	18 (14–26)	0.984	17 (14–24)	17 (14–26)	0.716
Reoperation, *n* (%)	4 (3.5%)	17 (3.1%)	0.811	4 (3.8%)	3 (1.4%)	0.227
30-day mortality, *n* (%)	0 (0%)	3 (0.5%)	0.569	0 (0%)	2 (1.0%)	0.554
90-day mortality, *n* (%)	1 (0.9%)	5 (0.9%)	0.726	1 (1.0%)	2 (1.0%)	1.000
POPF	14 (12.2%)	70 (12.6%)	0.908	14 (13.3%)	35 (16.7%)	0.442
Biochemical leak, *n* (%)	8 (7.0%)	35 (6.3%)	0.788	8 (7.6%)	21 (10.0%)	0.491
CR-POPF, *n* (%)	6 (5.2%)	35 (6.3%)	0.664	6 (5.7%)	14 (6.7%)	0.744
Grade B	4 (3.5%)	23 (4.1%)		4 (3.8%)	9 (4.3%)	
Grade C	2 (1.7%)	12 (2.2%)		2 (1.9%)	5 (2.4%)	
Biliary fistula, *n* (%)	6 (5.2%)	22 (3.9%)	0.536	6 (5.7%)	11 (5.2%)	0.860
Anastomotic fistula, *n* (%)	0 (0.0%)	2 (0.4%)	0.521	0 (0.0%)	0 (0.0%)	\
PPH, *n* (%)	2 (1.7%)	15 (2.7%)	0.554	2 (1.9%)	7 (3.3%)	0.723
Infection, *n* (%)	16 (13.9%)	84 (15.1%)	0.749	16 (15.2%)	33 (15.7%)	0.912
DGE, *n* (%)	4 (3.5%)	7 (1.3%)	0.214	4 (3.8%)	2 (1.0%)	0.098
Others, *n* (%)	8 (7.0%)	25(4.5%)	0.333	8 (7.6%)	11 (5.2%)	0.403
Total, *n* (%)	31 (27.0%)	141 (25.3%)	0.714	31 (29.5%)	58 (27.6%)	0.723

Others including complications such as pulmonary infection, deep venous thrombosis (DVT), liver abscess, cholangioenteric anastomotic stenosis and chyle leakage Clavien–Dindo Grade ≥ 3

*OPD* open pancreatoduodenectomy, *RPD* robotic-assisted pancreatoduodenectomy, *POD* postoperative days, *CR-POPF* clinical relevant postoperative pancreatic fistula (ISGPF grade B and C), *PPH* post pancreatectomy hemorrhage, *DGE* delayed gastric emptying

### DFS and OS

The median follow-up time was 18 (range: 2–74) months.

**Fig. 2:**
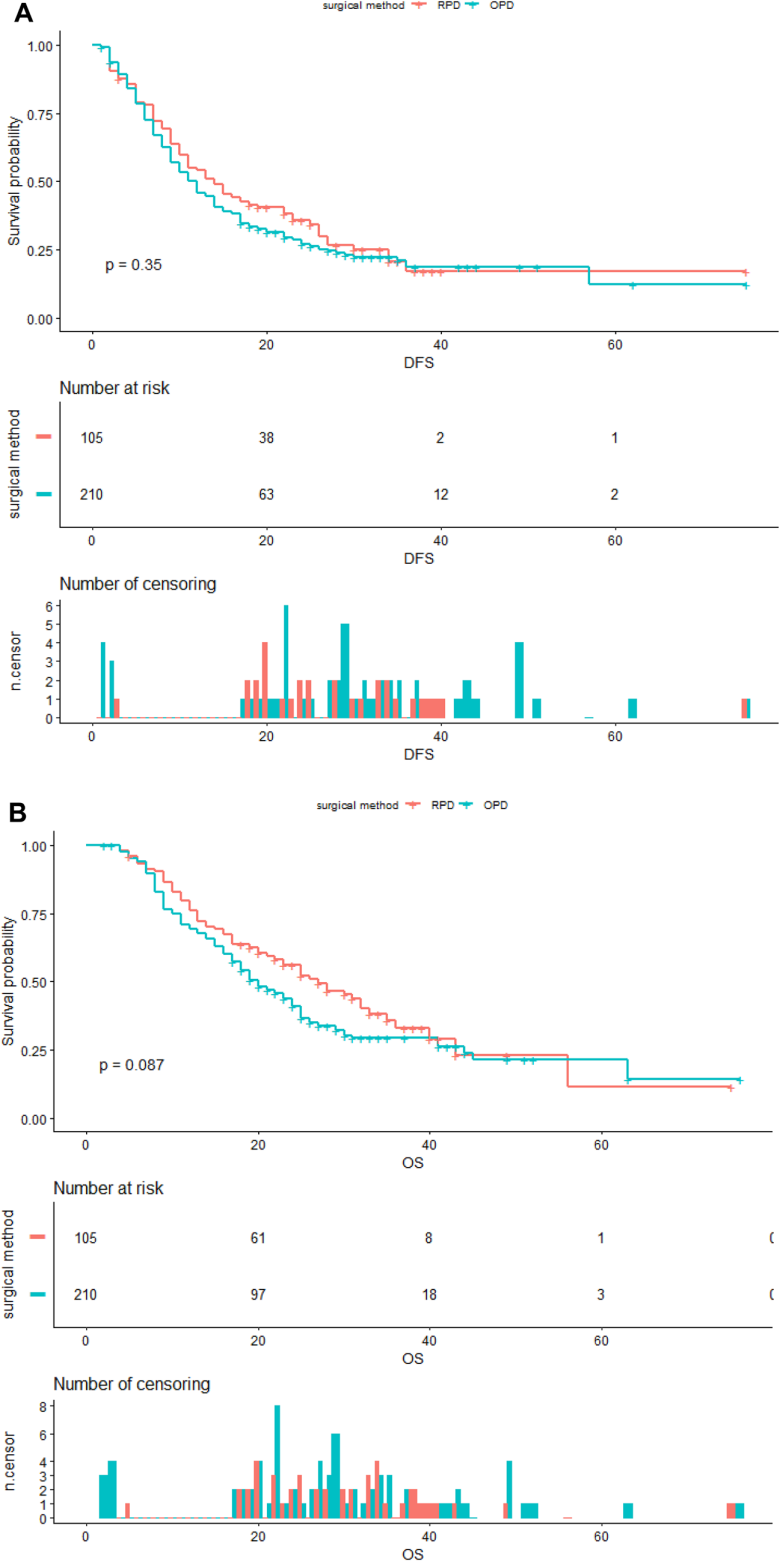
Kaplan–Meier curves of disease free survival (**A** log-rank test, *P* = 0.345) and overall survival (**B** log-rank test, *P* = 0.087) of propensity score matched cohorts

The Kaplan–Meier DFS and OS curves after matching are shown in Fig. 2.

The median DFS was 14 months (95% CI 11–22 months) in the RPD group and 12 months (95% CI 10–14) in the OPD group (HR 0.94; 95% CI 0.87–1.50; log-rank *P* = 0.345).

The median OS was 27 months (95% CI 22–35 months) in the RPD group and 20 months (95% CI 18–24) in the OPD group (HR 0.77; 95% CI 0.57–1.04; log-rank *P* = 0.087).

The 1-year DFS, 3-year DFS, 1-year OS, 3 year OS, the recurrence rate, recurrence patterns and post-recurrence chemotherapy rate are presented in Table 3.

**Table 3:** Outcomes of the propensity score matched population

Variables	Propensity score matched cohort		*P*
	RPD (*n* = 105)	OPD (*n* = 210)	
DFS			0.345
1-year DFS, (95% CI)	53.9% (45.1–64.4%)	45.9% (39.5–53.3%)	
3-year DFS, (95% CI)	17.1% (9.5–30.8%)	18.5% (13.1–26.1%)	
Recurrence, *n* (%)	62 (59.0%)	138 (65.7%)	0.587
Recurrence pattern, *n* (%)			
Local only	16 (25.8%)	41 (29.7%)	0.572
Liver metastasis only	15 (24.2%)	35 (25.4%)	0.860
Local and liver metastasis	16 (25.8%)	50 (36.2%)	0.147
Other	15 (24.2%)	12 (8.7%)	0.003
Post recurrence chemotherapy	43 (69.4%)	75 (54.3%)	0.046
OS			0.087
1-year OS, (95% CI )	76.0% (68.2–84.7%)	69.5% (63.4–76.1%)	
3-year OS, (95% CI )	33.0% (23.3–46.8%)	29.4% (23.1–37.3%)	

Other, include lung metastasis, bone metastasis, trocar or incision implantation metastasis and malignant ascites

*OPD* open pancreatoduodenectomy, *RPD* robotic-assisted pancreatoduodenectomy, *DFS* disease-free survival, *OS* overall survival

### COX regression analysis

Univariate analysis showed that BMI, CA199 ≥ 300 U/mL, operative time, estimated blood loss, tumor size, T3 stage and R1 resection were associated with DFS (*P* < 0.10) (Table 4). Multivariate analysis with COX regression model revealed the following independent predictors of DFS: BMI index (HR 0.94; 95% CI 0.90–0.98; *P* = 0.008), higher CA199 level (> 300 U/mL) (HR 1.52; 95% CI 1.14–2.03; *P* = 0.005), estimated blood loss (HR 1.00, 95% CI 1.00–1.01; *P* = 0.008) and T3 stage (HR 1.82, 95% CI 1.25–2.66; *P* = 0.002) (Table 4).

**Table 4:** Univariate and multivariate cox regression analysis of disease-free survival

Clinical variations	Univariate		Multivariate	
	HR (95% CI)	*P* value	HR (95% CI)	*P* value
Age	1.00 (0.99–1.02)	0.678		
Sex				
Male	Ref			
Female	0.99 (0.76- 1.30)	0.969		
BMI	0.95 (0.91–0.99)	**0.033**	0.94 (0.90–0.98)	**0.008**
CA199 (U/mL)				
< 150	Ref			
≥ 150, < 300	1.06 (0.72–1.55)	0.774	1.06 (0.72–1.56)	0.759
≥ 300	1.49 (1.12–1.99)	**0.006**	1.52 (1.14–2.03)	**0.005**
Operative time	1.00 (1.00–1.00)	**0.044**	1.0008(0.999–1.003)	0.463
Estimated blood loss	1.00 (1.00–1.00)	**0.005**	1.00 (1.00–1.01)	**0.008**
Tumor size	1.19 (1.06–1.33)	**0.003**	1.06 (0.91–1.23)	0.472
PV/SMV reconstruction				
No	Ref			
Yes	1.25 (0.81–1.93)	0.312		
T stage				
T1	Ref			
T2	1.3455 (0.94–1.93)	0.106	1.26 (0.87–1.81)	0.221
T3	1.93 (1.34–2.80)	**< 0.001**	1.82 (1.25–2.66)	**0.002**
N stage				
N0	Ref			
N1	0.98 (0.74–1.30)	0.902		
N2	1.10 (0.69–1.76)	0.688		
Nodal status				
N−	Ref			
N+	1.00 (0.77–1.30)	0.971		
Examined lymph node				
≤ 8	Ref			
> 8	1.13 (0.87–1.48)	0.356		
LNR				
< 0.1	Ref			
≥ 0.1	1.05 (0.78–1.40)	0.754		
Differentiation				
Well-intermediate	Ref			
Poor	1.13 (0.84–1.53)	0.425		
Margin				
R0	Ref			
R1	1.40 (0.94–2.08)	**0.097**	1.27 (0.85–1.90)	0.246
LVI				
Negative	Ref			
Positive	1.19 (0.92–1.54)	0.190		
PNI				
Negative	Ref			
Positive	0.96 (0.72–1.29)	0.789		
Adjuvant chemotherapy				
No	Ref			
Yes	1.08 (0.84–1.40)	0.538		
Surgery				
OPD	Ref			
RPD	0.88 (0.67–1.16)	0.359	0.90 (0.68–1.19)	0.456

Univariable associations (*P* < 0.10) that were selected for multivariable analysis and significant factors (*P* < 0.05) on multivariable analysis are shown in bold

Univariate analysis showed that BMI, CA199 ≥ 300 U/mL, estimated blood loss, tumor size, T3 stage, lymphovascular invasion (LVI) and type of surgery were associated with OS (*P* < 0.10) (Table 5). Multivariate analysis with COX regression model revealed the following independent predictors of OS: BMI index (HR 0.94; 95% CI 0.90–0.99; *P* = 0.012), high CA199 level (> 300 U/mL) (HR 1.56, 95% CI 1.15–2.13; *P* = 0.005), estimated blood loss (HR 1.00, 95% CI 1.00–1.00; *P* = 0.008) and T3 stage (HR 1.71, 95% CI 1.15–2.54; *P* = 0.008) (Table 5).

**Table 5:** Univariate and multivariate cox regression analysis of overall survival

Clinical variations	Univariate		Multivariate	
	HR (95% CI)	*P* value	HR (95% CI)	*P* value
Age	1.00 (0.99–1.02)	0.824		
Sex				
Male	Ref			
Female	0.94 (0.70–1.26)	0.680		
BMI	0.95 (0.91–1.00)	**0.057**	0.94 (0.89–0.99)	**0.012**
CA199 (U/mL)				
<150	Ref			
≥ 150, < 300	1.03 (0.67–1.56)	0.907	1.06 (0.70–1.61)	0.785
≥ 300	1.48 (1.09–2.01)	**0.013**	1.56 (1.15–2.13)	**0.005**
Operative time	1.00 (1.00–1.00)	0.114		
Estimated blood loss	1.00 (1.00–1.00)	**0.006**	1.00 (1.00–1.00)	**0.008**
Tumor size	1.17 (1.03–1.32)	**0.015**	1.08 (0.92–1.25)	0.355
PV/SMV reconstruction				
No	Ref			
Yes	1.36 (0.84–2.19)	0.208		
T stage				
T1	Ref			
T2	1.14 (0.77–1.67)	0.510	1.14 (0.77–1.68)	0.514
T3	1.79 (1.21–2.64)	**0.003**	1.71 (1.15–2.54)	**0.008**
N stage				
N0	Ref			
N1	1.09 (0.81–1.48)	0.559		
N2	0.98 (0.58–1.66)	0.951		
Nodal status				
N−	Ref			
N+	1.07 (0.81–1.42)	0.634		
Examined lymph node				
≤ 8	Ref			
> 8	1.21 (0.90–1.63)	0.201		
LNR				
< 0.1	Ref			
≥ 0.1	1.07 (0.78–1.46)	0.687		
Differentiation				
Well-intermediate	Ref			
Poor	0.96 (0.69–1.32)	0.786		
Margin				
R0	Ref			
R1	1.24 (0.80–1.91)	0.341		
LVI				
Negative	Ref			
Positive	1.35 (1.01–1.79)	**0.040**	1.45 (1.09–1.95)	**0.012**
PNI				
Negative	Ref			
Positive	1.01 (0.73–1.39)	0.953		
Adjuvant chemotherapy				
No	Ref			
Yes	1.05 (0.79–1.38)	0.743		
Surgery				
OPD	Ref			
RPD	0.77(0.57–1.04)	**0.094**	0.77(0.57–1.05)	0.094

Univariable associations (*P* < 0.10) that were selected for multivariable analysis and significant factors (*P* < 0.05) on multivariable analysis are shown in bold

Surgery type (RPD *vs.* OPD) was not a significant predictor in DFS (HR 0.90; 95% CI 0.68–1.19; *P* = 0.456) and OS (HR, 0.77; 95% CI 0.57–1.05; *P* = 0.094).

## Discussion

Since first reported 16 years ago [6], RPD was adopted in pancreatic head, biliary duct and periampullar tumors by some high-volume centers, and its intraoperative and perioperative outcomes was acceptable [4, 12, 30, 31]. Recent studies showed robotic cervical cancer surgery was associated with lower rates of DFS and OS [32, 33], which cause suspicion about the oncological outcomes of other robotic surgery for cancer. In this high volume single-center retrospective study using the propensity score-matching method, we compared the DFS and OS of RPD *versus* OPD in PDAC patients. Our study demonstrated that patients receiving RPD had comparable DFS and OS *versus* those undergoing OPD.

Before matching, the RPD group and OPD group differed significantly in previous abdominal surgery history, total bilirubin, year of diagnosis and lymphovascular invasion rate. These may be explained as follows: (1) Surgeons’ preference and suggestion of RPD or OPD, to some extent, was determined by patients’ total bilirubin index and abdominal surgery history; (2) patients’ acceptance of robotic-assisted surgery and proficiency of surgeons in different periods could influence the proportion of patients receiving RPD in different years. However, these differences disappeared after propensity score matching and the other baseline characteristics gained further equivalence to reduce the patient-selection bias.

A number of studies about the comparison between RPD and OPD had been published [4, 11, 12, 34], some previous studies showed that main complications such as postoperative pancreatic fistula, biliary fistula, infection and postoperative hemorrhage were not significantly different between minimally invasive pancreatoduodenectomy and OPD [4], some studies even hold a positive attitude towards perioperative short-term outcomes of RPD. In this study, the onset of main complications together with other complications were comparable between the two cohorts. Meanwhile, the estimated blood loss and operative time, which were associated with surgical trauma, were also similar in the two cohorts. The postoperative days, reoperation rate, 30-day and 90-day mortality rate also showed no statistical difference between the two cohorts, prompting us to conclude that the perioperative outcomes of RPD were comparable to those of OPD for PDAC patients. Nevertheless, we cannot neglect that compared with our previous study of RPD [23], when applied in PDAC patients, RPD showed no superiority in perioperative outcomes, which need more samples and specific data collection and analysis on surgical-related prognosis.

The principal object of this study was to compare the oncological outcomes of RPD and OPD for PDAC, and the concerns on the oncological safety of RPD for PDAC can be described as the worries about the ability to have R0 resection and adequate lymphadenectomy in RPD. After matching, variables that might influence DFS and OS such as the number of retrieved and positive lymph nodes, lymph node ratio, lymphovascular invasion and perineural invasion, R0 resection rate, tumor differentiation, T stage, N stage, as well as the postoperative adjuvant chemotherapy rate showed no significant difference between the two cohorts, suggesting that the RPD was equally effective in pathological level when compared with OPD. In this study, R0 resection rate in RPD group was 88.6%, not significantly different from that (89.0%) in OPD group, besides the pancreatic neck margin, uncinate process and retroperitoneal margins are that surgeons always pay attention to, which means “vascular margin” should not be neglected. There was a remarkable proportion of PV/SMV involvement cases in this study. To achieve R0 resection, PV/SMV resection and reconstruction were routinely performed, including wedge resection and repair, end-to-end anastomosis primarily or with gortex grafts. Studies focusing on vascular resection of RPD also showed that vein resection had acceptable perioperative risks and could achieve better survival outcome than that without vein resection [35, 36], and PV/SMV involvement was not a contraindication in RPD for PDAC.

Lymphadenectomy was also a crucial point in curative surgery for PDAC patients. The protocol of lymphadenectomy we used in this study depended on the range of Japan Pancreas Society standard lymphadenectomy [37]. In OPD, with tactile sensation, we had better way to explore the positive suspected lymph nodes, and resect these positive suspected lymph nodes subsequently, which eventually induced more advanced N stage and more positive lymph nodes in the OPD cohort before matching. Compared with OPD, RPD had a different operation view and different approach to perform lymphadenectomy, but with a standard protocol, RPD showed no disadvantages in lymphadenectomy when compared with OPD cohort after matching.

With similar perioperative oncological outcomes and similar adjuvant chemotherapy rate, the Kaplan–Meier survival curve and stratified log-rank test showed that the two cohorts achieved similar DFS (*P* = 0.345). We found three common recurrence patterns in this study, which were local–regional only, liver metastasis only, simultaneous local–regional and liver metastasis, the proportions of these three recurrence patterns were comparable in the two cohorts. Other rare types of recurrence showed difference between the two cohorts, 5(8.1%) trocar implantation, 7 (11.3%) lung metastasis, 1 (1.6%) bone metastasis, 2 (3.2%) malignant ascites were found in RPD cohort, and 4 (2.9%) incision implantation, 7 (5.1%) lung metastasis, 1 (0.7%) malignant ascites were found in OPD groups. The different post-recurrence chemotherapy rate was an important factor to influence the OS (RPD: 27 [95% CI 22–35] *vs.* OPD: 20 [95% CI 18–24] months (HR 0.77; 95% CI 0.57–1.04; log-rank *P* = 0.087), patients in RPD cohorts seemed to have better acceptance and tolerance to receive post-recurrence chemotherapy, further studies should be designed to verify the connection between minimally invasive pancreatoduodenectomy and post-recurrence survival.

To investigate survival predictors, we established univariate and multivariate Cox regression analyses model to find out variables associated with DFS and OS. Unadjusted factors significantly associated with DFS and OS (those with *P* value < 0.1), were analyzed by multivariable Cox regression model, and BMI, high CA 19–9 level, estimated blood loss and T3 stage proportion were the independent predictors of both DFS and OS, while LVI was an independent risk factor of OS. No significant difference was observed with COX regression model in DFS and OS between RPD and OPD cohorts. These survival analysis results helped us to draw a conclusion that in curative surgery for treatment-naïve PDAC patients, RPD had a non-inferior effect on DFS and OS when compared with OPD.

The study has several limitations. Although the propensity score matching method increased the reliability and credibility, the inevitable selection bias of retrospective study still existed. In addition, due to missing data and loss to follow-up, some cases were eliminated, which led to the reduction in sample capacity, and the limitation of follow-up period does not allow us to get the complete follow-up data such as the 5-year survival rate. Thirdly, according to our recent study, an important flexion point of RPD learning curve was case No. 250, however considered that excluding the cases in learning curve may cause insufficiency number of cases and follow-up time, we collected cases after the first flexion point of learning curve according to a previous study [20]. Also, neoadjuvant chemotherapy for resectable PDAC was applied in some high-volume center for better survival outcomes, but was not applied in our center before 2018, so oncological outcomes of RPD *vs.* OPD for PDAC patients after neoadjuvant chemotherapy still need further analysis. At last, as for sample quantity, the data source was a high volume single center, though with quality control, still not comparable to multicenter studies.

## Conclusions

RPD was comparable to OPD in surgical safety and feasibility, perioperative prognosis, oncological outcomes, and, most importantly, survival outcomes. This study provides important evidence supporting the utilization of RPD in PDAC patients. Based on our work, further prospective randomized controlled studies should be planned to verify the oncological and survival outcomes of RPD in PDAC patients.
